# The effect of exercise training on left ventricular function in young elite athletes

**DOI:** 10.1186/1476-7120-9-27

**Published:** 2011-10-12

**Authors:** Alessio De Luca, Laura Stefani, Gianni Pedrizzetti, Stefano Pedri, Giorgio Galanti

**Affiliations:** 1 Sport medicine Centre, University of Florence, V. delle Oblate, 4, 50134 Florence, Italy; 2 D.I.C.A. University of Trieste, Piazzale Europa, 1, 34127 Trieste, Italy

**Keywords:** strain, twist, multilayer, endocardium, epicardium, athletes, sport

## Abstract

**Background:**

Regular training, in particular endurance exercise, induces structural myocardial adaptation, so-called "athlete's heart". In addition to the 2D standard echo parameters, assessment of myocardial function is currently possible by deformation parameters (strain, rotation and twist). Aim of study is to assess the role of rotation and twist parameters for better characterize the heart performance in trained elite young athletes from different kind of sports. Eventually, verify early on any possible impact due to the regular sport activity not revealed by the standard parameters.

**Methods:**

50 young athletes (16 cyclists, 17 soccer players, 17 basket players) regularly trained at least three times a week for at least 9 months a year and 10 young controls (mean age 18.5 ± 0.5 years) were evaluated either by to 2D echocardiography or by a Speckle Tracking (ST) multi-layer approach to calculate Left Ventricle (LV) endocardial and epicardial rotation, twist, circumferential strain (CS) and longitudinal strain (LS). Data were compared by ANOVA test.

**Results:**

All the found values were within the normal range. Left Ventricle Diastolic Diameter (LVDD 51.7 ± 2.6 mm), Cardiac Mass index (CMi 114.5 ± 18.5 g/m^2^), epi-CS, epi-LS, epicardial apex rotation and the Endo/Epi twist were significantly higher only in cyclists. In all the groups, a physiological difference of the Endo/Epi basal circumferential strain and twist values have been found. A weak but not significant relationship between the Endo and twist values and LVDD (r^2 ^= 0.44, p = .005) and CMi was also reported in cyclists.

**Conclusions:**

Progressive increase of apical LV twist may represent an important component of myocardial remodelling. This aspect is particularly evident in the young cyclists group where the CMi and the LVDD are higher. ST multilayer approach completes the LV performance evaluation in young trained athletes showing values similar to adults.

## Background

Regular sport activity normally induces myocardial structural and functional modifications [1-3]. Walls thickness and LV chamber dimensions are particularly involved in this adaptation.

It can typify different morphological heart features in consequence to various (pressure and/or volume overload) kinds of practiced sports [4]. These characteristics are currently assessed in athletes by 2D standard echocardiographic parameters (CMi, chamber diameters, wall thickness).

Cardiac functional parameters based on myocardial "deformation", are currently used to investigate the heart's function with high sensibility and specificity [5-8].

Investigations in elite athlete's heart have recently provided relevant information on the apex myocardial "reserve" of the left ventricle (LV) [9-13]. However, myocardial adaptation to regular training in young elite athletes up until now has not been studied and very little informations about the LV deformation parameters in young athletes are available.

Aim of the study is to assess the myocardial structure and function in three different regularly trained groups of young elite athletes (basket, soccer and cycling), by ST multi-layer approach.

## Methods

### Study population

A group of 50 male young competitive athletes (16 cyclists, 17 soccer players, 17 basket players) and 15 male young controls (mean age 18.5 ± 0.5 yrs) were enrolled. The study was approved by local Ethics Committee and an oral informed consent was obtained before the participation to the protocol. For subjects under 18 yrs of age, parental consent was also necessary. Exclusion criteria included evidence of cardiovascular and metabolic disease whose presence had been confirmed by the clinical history. Although it is not a mandatory exam in order to obtain the elegibility, all subjects underwent to echocardiographic exam at rest, including the evaluation of the 2D and deformation parameters. Additional images were recorded in order to permit the post processing analysis by dedicated software.

### Training regimens

All the athletes were enrolled during the routine evaluation for agonistic sport elegibility. They were regularly trained: soccer and basketball players for 5 ± 1.2 hours/week for 6.7 ± 2.5 years vs. 4.5 ± 0.5 hours/week for 5.9 ± 2.3 years respectively, cyclists for 20.5 ± 0.5 hours/week for 7.4 ± 0.5 years. Controls were sex and age-matched. Although they trained for less than 100 minutes per week and they do not therefore practiced any kind of regular exercise for at least six months, if compared to athletes, controls were similar in anthropometric characteristics.

### Echocardiographic examination

All the echocardiographic exams were performed by two experienced board-certified cardiologists. The two cardiologists had worked together for more than 5 years, and no formal reliability of the studies (inter- or intra-tester) was done. However, in order to verify the substantial overlapping of the measurement performed, 5 subjects among the cases studied were randomly selected and blindly re-analyzed for the main 2D and deformation parameters considered.

From the long axis view, and following the AHA echo- guidelines [14], the standard LV 2D parameters were obtained at rest. The basal 2D systo-diastolic and Doppler parameters, inter ventricular septum (IVS) and posterior wall (PW) thickness, left ventricle end-diastolic diameter (LVEDd), left ventricle end-systolic diameter (LVESd), left atrium (LA) and aortic root (Ao) dimensions, pulse wave Doppler transmitral flow E-wave, A-wave (E/A ratio), deceleration time (DT), isovolumic relaxation time (IVRT) were calculated. The diastolic function study was also completed analyzing the E/E' ratio calculation by the Tissue Doppler Imaging (TDI) method [7] in order to better classify the associated diastolic pattern. The evaluation of left ventricular Cardiac Mass Index g/m^2 ^(CMI) was obtained from Devereux procedure [15], and the EF % was calculated as (EDV-ESV)/EDV, where EDV and ESV are the LV end-diastolic and end-systolic volumes, respectively. The degree of the severity of the valve insufficiency, described as the extent of the regurgitating jet on a 0 to 4+ scale, was assessed using the color-flow mapping method from the four-chamber view according to the AHA echo- guidelines [14].

### Image acquisition

**Figure 1:**
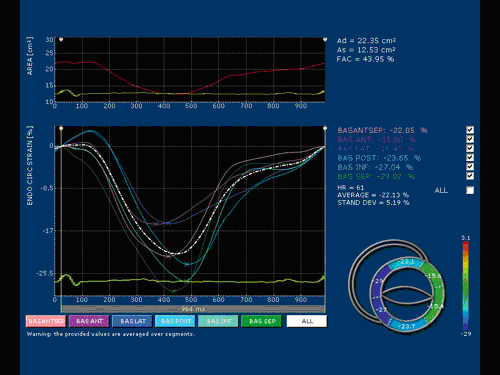
**Example of endocardial circumferential strain at basal level of LV assessed by X-Strain software (ESAOTE, Italy)**.

**Figure 2:**
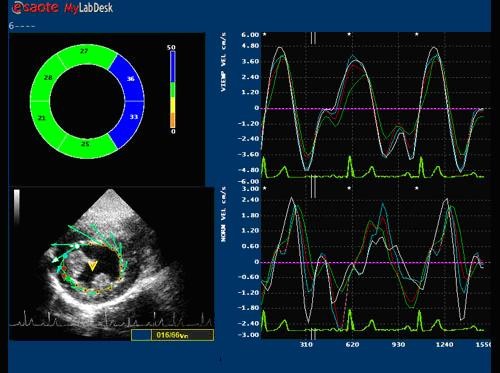
**Example of endocadial basal rotation of LV assessed by X-Strain Software (ESAOTE, Italy)**.

Images were obtained in the left lateral decubitus position, after a 15 minute rest using the Echocardiograph My Lab 50 (ESAOTE, Florence, Italy) equipped with a 2.5 MHz probe. The two-dimensional grey scale images were obtained at a frame rate between 60 and 70 Hz. The images were acquired in a cine loops triggered to the QRS complex, in a resting condition and at a heart rate (HR) of approximately 70 bps to avoid the effects of a large HR variability on the rotation and the twist. High quality images were acquired with excellent visualization of the endocardial and epicardial borders. Acquisitions of the apical and basal segments were stored digitally for subsequent off-line analysis with dedicated ST software included in the echocardiograph as shown in the Figure 1 and 2.

### LV Circumferential strain, rotation and torsion by Speckle Tracking method

Speckle tracking is a novel and under scrutiny method for the assessment of myocardial deformation [16-18]. It is suitable both for endocardial and epicardial layer (multilayer approach). For each subject studied, two echocardiographic images of the LV chamber from the short-axis view at the mitral and apex levels were obtained. The short axis images were captured taking care to ensure that the basal plane was contained the mitral valve and that the apical plane was acquired distally to the papillary muscle. Particular care has been taken acquiring these views. It is known that the measurement of LV apical rotation is critically dependent on transducer position. Therefore, as literature reports [18], the standard parasternal long-axis view was defined as the position in which the LV and aorta were most inline, with the mitral valve tips in the middle of the sector. From that window an as-circular-as-possible short-axis image of the LV apex, just proximal to the level with end-systolic LV luminal obliteration, was obtained by angulation of the transducer. The short-axis views, captured at the mitral valve plane and apex level, were subsequently processed by the speckle tracking X-Strain software. The software asks the operator to give the initial position of the tracking points. The system used provides two different ways for the first tracking point insertion: free-hand mode where there's no limits to the number and location of the tracking points or assisted mode (AHS™, Aided Heart Segmentation). The later mode assists the operator in inserting well equal-spaced tracking points over the 2D images and it is based on ASE 18 segments segmentation of the heart [19]. AHS method has been used in our protocol to reduce measures variability among operators. Following AHS™, 12 tracking points were superimposed at the end of the diastole on the echocardiographic image at basal level of the LV chamber and 8 tracking points were positioned in apex. The tracking of each point over the time is then performed with a 2D technique. A square window of 16 pixels on each side of the current position of the point is extracted from all B-Mode frames, then frame by frame displacement is computed both in horizontal and in vertical directions as to minimize total brightness convention error. Considering the regular LV geometry and also for the complete absence of the myocardial synergic dissimilarities of the population studied, the results were accepted in averaged values, with indication of the standard deviation. The parameters evaluated were Circumferential Strain (ε-circum %), Radial Strain, Endocardial and Epicardial Rotation. Twist has been therefore calculated from a post processing analysis as the net difference of LV mean rotation between basal and apical short-axis plane for each subject. This is more properly a differential rotation, although the homogeneity of the population, and the small variability in the LV size, make results equivalent to those evaluable from actual twist, apical and basal rotation.

### Statistical analysis

All the data were reported as mean ± SD. Statistical analysis was possible by SPSS 17.0 software. Two-way ANOVA with post-hoc analysis by LSD to compare the groups was used. Multivariate correlations among Endo/Epi twist and indexed cardiac mass (CMi), ejection fraction (EF), heart rate (HR), left ventricle diastolic diameter (LVDD) left ventricle systolic diameter (LVSD) and age were performed. A *p *value ≤ 0.05 was considered statistically significant.

## Results and discussion

### 2D-echocardiographyc exam

All the general data were reported in Table 1. The results show a little but not significant increase of mean heart rate values in cyclists. Although the heart systolic function, expressed as EF, resulted to be substantially similar among the athletes, controls showed significantly lower values compared to basket players (61.4±1.3 vs. 65.6±5.1%; p <.01) and soccer players (65.6±4.6%; p = .01). Concerning the standard morphological echo parameters, LV systolic and diastolic diameters were higher especially in cyclists respect to controls.

**Table 1:** Standard 2D-echocardiography data of the three groups of athletes

	Cycling	Soccer	Basket	Controls
**Age (yrs)**	17.6 ± 0.5	16.5 ± 2.5	17.0 ± 2.5	22.8 ± 3.2^∞^
**Heart rate (bpm)**	76.4 ± 11.5	71.05 ± 13.4^¶^	81.82 ± 12.4	73.2 ± 8.9
				
**LV parameters**				
**IVS (mm)**	9.47 ± 0.9	9.38 ± 1.0	9.16 ± 0.9	8.92 ± 0.9
**PW(mm)**	9.49 ± 0.8	9.25 ± 0.7	8.93 ± 0.9	8.76 ± 1.0^#^
**LVDD (mm)**	51.7 ± 2.6	50.2 ± 4.0	50.5 ± 3.6	47 ± 3.1^♥ Δ ¶^
**LVSD (mm)**	33.6 ± 1.7	31.9 ± 3.1	32.0 ± 3.3	30.2 ± 2.2^♦^
**CMi (gr/m^2^)**	114.5 ± 18.5^¶^	105.9 ± 16.9	100.7 ± 14.3	87.4 ± 9.8^£ * $^
**WRT**	0.36 ± 0.05	0.37 ± 0.06	0.35 ± 0.05	0.38 ± 0.07
**EF (%)**	63.8 ± 2.5	65.6 ± 4.6	65.6 ± 5.1	61.4 ± 1.3^Ω Φ^
				
**LV diastolic function**				
**E wave (m/s)**	79.2 ± 14.5	97.1 ± 11.7^£^	95.9 ± 15.3^♥^	86.4 ± 13.2
**A wave (m/s)**	42.9 ± 6.7^§^	49.0 ± 17.5	53.0 ± 11.4^♦^	58.4 ± 12.5
**DT (sec.)**	158.8 ± 12.4	157.5 ± 18.9	175.1 ± 24.1	160.4 ± 54
**IVRT (sec.)**	70.7 ± 8.7	70.2 ± 7.1	66.3 ± 8.7	84 ± 26.4^∞^

Legend: **IVS: interventricular septum, PW: posterior wall, LVDD: left ventricle diastolic diameter, LVSD: left ventricle systolic diameter, EF: ejection fraction, CMi: cardiac mass index, WRT: wall ratio thickness, DT: deceleration time, IVRT: isovolumic relaxation time.**

Significant differences: *p = .004 vs. soccer, ^§^*p *= .001 vs. controls, ^♦^*p *= .03 vs. cycling, ^∞^*p *< .001 vs. other groups, ^♥^*p *= .001 vs. cycling, ^Δ^*p *= .02 vs. soccer; ^¶^*p *= .01 vs. basket; ^£^*p *< .001 vs. cycling, ^$^*p = *.04 vs. basket; ^Ω^*p *= .01 vs. soccer;^Φ^*p *< .01 vs. basket; ^¥^*p *= .006 vs. basket, ^‡^*p *= .007 vs. controls; ^#^*p *= .05 vs. controls.

The CMi was significantly increased more in cyclists in comparison to basket players (114.5 ± 18.5 vs. 100.7 ± 14.3 gr/m^2^; p = .01) and controls (87.4 ± 9.8 gr/m^2^; p <.001). At the same time a significantly lower CMi and LV diameters were found in controls and not in the athletes. The wall ratio thickness (WRT) resulted to be similar among the groups. Concerning diastolic function, IVRT was significantly higher in controls than other groups, while DT was homogenous among the athletes. E and A waves were in general significantly lower in cyclist compared to other groups.

### Endo/Epi regional LV longitudinal, circumferential strain, rotation and twist

**Figure 3:**
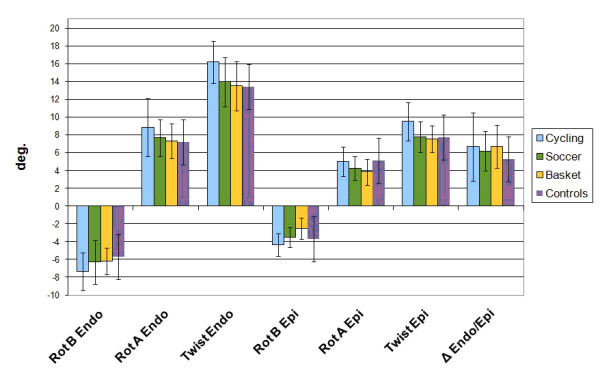
**LV rotation and twist histogram for epicardial and endocardial layers**.

Mean strain, rotation and twist values differences were appreciated among the groups at rest and all results are reported in Table 2 and Figure 3. The epicardial longitudinal peak systolic strain was significantly lower in cyclists (-11.70 ± 2.0%) than other athletes (-13.43 ± 2.4%; p = .04 vs. soccer players, -13.72 ± 2.9%; p = .03 vs. basket players). Mean epicardial apical circumferential peak systolic strain, therefore, was significantly increased in cyclists compared to soccer players (-15.55 ± 4.7 vs. -12.75 ± 3.9%; p = .04) and in basket players compared to controls (-11.06 ± 3.2 vs. -14.56±6.5%; p = .05). LV basal epicardial rotation was lower in controls in respect to other groups. It was also higher in cyclists (-4.34 ± 1.2°) than in soccer players (-3.52 c 1.1°; p = .04). Epicardial apical rotation was lower in basket players (3.81 ± 1.5°) than cyclists (5.0 ± 1.7°; p = .04) and controls (5.10±2.3; p = .05). Consequently, LV epicardial twisting resulted significantly increased in cyclists in respect to other groups. Concerning endocardial deformation parameters, only basal rotation (-7.32±2.1 vs. -5.69±1.0; p = .04) and apical circumferential strain (-37.64±8.5 vs. -29.49±7.4; p = .02) were significantly higher in cyclists compared to controls respectively. However, endocardial twist was significantly higher in cyclists than in any of the other groups. All the control group parameters were significantly lower in respect to cyclists, while the difference of this group in respect to the others was only significant for the mean rotation of basal LV segments.

**Table 2:** Peak systolic strains, rotation and twist of subendocardial and subepicardial layers among the three groups of athletes

	Cycling	Soccer	Basket	Controls
**Endocardial parameters**				
**Mean circum-ε (%)**				
**Basal**	-23.02 ± 4.6	-23.40 ± 2.6	-21.82 ± 3.5	-23.63 ± 3.5
**Apical**	-37.64 ± 8.5^¥^	-33.82 ± 8.0	-32.15 ± 9.3	-29.49 ± 7.4
**Mean Longit- ε (%)**	-16.72 ± 3.2	-17.84 ± 2.8	-17.96 ± 2.9	-18.08 ± 2.3
**Mean Rotation (°)**				
**Basal**	-7.32 ± 2.1^£^	-6.29 ± 2.4	-6.19 ± 1.5	-5.69 ± 1.0
**Apical**	8.86 ± 3.2	7.65 ± 2.1	7.29 ± 2.0	7.79 ± 1.8
**Mean Twist (°)**	16.18 ± 4.1^* Φ ?^	13.94 ± 2.7	13.48 ± 3.4	12.89 ± 2.4
				
**Epicardial parameters**				
**Mean circum- ε (%)**				
**Basal**	-13.50 ± 3.3	-14.53 ± 2.5	-13.87 ± 2.6	-13.28 ± 1.6
**Apical**	-15.55 ± 4.7^*^	-12.75 ± 3.9	-11.06 ± 3.2^Δ^	-14.56 ± 6.5
**Mean Longit- ε (%)**	-11.70 ± 2.0^¶ **†**^	-13.43 ± 2.4	-13.72 ± 2.9	-14.73 ± 3.3
**Mean Rotation (°)**				
**Basal**	-4.34 ± 1.2^* ♥^	-3.52 ± 1.1^∞^	-3.71 ± 1.2^?^	-2.53 ± 0.7
**Apical**	5.00 ± 1.7^♦^	4.26 ± 1.3	3.81 ± 1.5^Δ^	5.10 ± 2.3
**Mean Twist (°)**	9.51 ± 2.4^**# § **¥^	7.78 ± 1.7	7.52 ± 1.5	7.63 ± 2.1
**Δ Endo/Epi**	6.67 ± 3.8	6.17 ± 2.2	5.96 ± 2.4	5.48 ± 1.47

Legend:. *p = .04 vs. soccer; ^†^*p *= .03 vs. basket; ^♦^*p *= .04 vs. basket; ^‡^*p *= .006 vs. basket; ^#^*p *= .01 vs. soccer; ^Φ^*p *= .02 vs. basket, ^§^*p *= .005 vs. basket, ^£^*p = *.04 vs. controls, ^¥^*p *= .02 vs. controls, ^?^*p *= .01 vs. controls, ^∞^*p *= .03 vs. controls, ^♥^*p *< .001 vs. controls, ^Δ^*p *= .05 vs. controls, ^¶^*p *= .005 vs. controls. **Circum-ε: circumferential strain; Longit-ε: longitudinal strain.**

### Segmental longitudinal strain gradient

As reported in Table 3, a base-to-apex longitudinal strain gradient was investigated. In cyclists, a gradient was evident particularly in the septal segment involving the basal and apical (-14.96 ± 5.0 vs. -19.83 ± 6.4%, p = .02) regions and also the medium apical ones (-15.40 ± 3.9%, p = .02). This behavior was evident also in the lateral wall, but significantly recognized only at the medium-apical segments (-14.81 ± 4.8 vs. -19.35 ± 5.6%, p = .02).

In soccer players, substantial and significant differences were found among all the segments of the septum (-14.71 ± 4.6 vs. -20.78 ± 5.4%, p = .001; basal-medium -17.43 ± 2.9, p = .03; and medium-apex p = .05).

**Table 3:** Segmental LV longitudinal peak systolic strain in all considered groups

		Basal	Medium	Apical	Bas-Ap P	Bas-Med P	Med-Ap P
**Endocardial parameters**							
**Cycling**	**Septum**	-14.96 ± 5.0	-15.40 ± 3.9	-19.83 ± 6.4	**.02**	**N.S.**	**.02**
	**Lateral wall**	-16.00 ± 5.5	-14.81 ± 4.8	-19.35 ± 5.6	**N.S.**	**N.S.**	**.02**
**Soccer**	**Septum**	-14.71 ± 4.6	-17.43 ± 2.9	-20.78 ± 5.4	**.001**	**.05**	**.03**
	**Lateral wall**	-16.94 ± 4.5	-18.55 ± 3.4	-18.66 ± 6.4	**N.S.**	**N.S.**	**N.S.**
**Basket**	**Septum**	-15.44 ± 4.2	-16.30 ± 3.0	-20.59 ± 6.8	**.01**	**N.S.**	**<.01**
	**Lateral wall**	-17.97 ± 4.8	-18.74 ± 3.8	-18.74 ± 6.7	**N.S.**	**N.S.**	**N.S.**
**Controls**	**Septum**	-23.72 ± 3.7	-29.44 ± 3.5	-15.88 ± 4.0	**<.001**	**N.S.**	**.004**
	**Lateral wall**	-17.10 ± 10.6	-23.0 ± 6.9	-16.91 ± 8.6	**N.S.**	**N.S.**	**N.S.**
**Epicardial parameters**							
**Cycling**	**Septum**	-11.33 ± 3.4	-12.54 ± 3.4	11.69 ± 6.0	**N.S.**	**N.S.**	**N.S.**
	**Lateral wall**	-11.67 ± 5.6	-12.08 ± 4.2	-10.88 ± 4.8	**N.S.**	**N.S.**	**N.S.**
**Soccer**	**Septum**	-13.22 **± **4.1	-15.44 ± 3.0	-13.05 ± 4.6	**N.S.**	**N.S.**	**N.S.**
	**Lateral wall**	-13.27 ± 4.9	-14.66 ± 3.5	-10.96 ± 4.1	**N.S.**	**N.S.**	**.01**
**Basket**	**Septum**	-15.92 ± 4.3	-15.45 ± 2.8	-11.95 ± 3.8	**.01**	**N.S.**	**<.01**
	**Lateral wall**	-14.93 ± 7.0	-12.77 ± 7.9	-11.32 ± 3.6	**N.S.**	**N.S.**	**N.S.**
**Controls**	**Septum**	-12.24 ± 5.0	-15.80 ± 2.8	-15.48 ± 3.7	**N.S.**	**N.S.**	**N.S.**
	**Lateral wall**	-14.74 ± 8.4	-16.50 ± 8.4	- 10.93 ± 5.9	N.S.	N.S.	N.S.

In basket players a similar behavior was found in the same region of the LV chamber (at septal basal-apex -15.44 ± 4.2 vs. -20.59 ± 6.8%, p = .01; medium-apex -16.30 ± 3.0%, p = .02)

The epicardial longitudinal strain value, resulted to be significantly lower only at LV septal level in basketball players (basal-apex -15.92 ± 4.3 vs. -11.95 ± 3.8%, p = .01; medium-apex -15.45 ± 2.8% vs. -11.95 ± 3.8%, p < .01) and at the LV lateral wall medium-apical segments of the soccer players (-14.66 ± 3.5 vs. -10.96 ± 4.1%, p = .01). On the contrary, no difference in cyclists was found. In controls, none significant variation of LV apical segments has been appreciated, while a higher deformation of basal LV segments was present.

### Correlations

**Figure 4:**
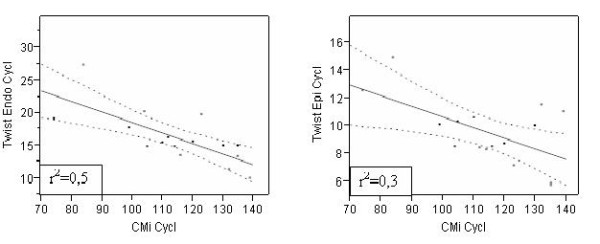
**Correlation CMi/twist in cyclists**.

No correlations among Endo/Epi twist values and HR, EF and age respectively were found among the groups. However a weak positive correlation among CMi and Endo/Epi twist only in cyclists, with an higher CMi than others, has been shown (Figure 4). A positive trend toward a correlation between LVDD and Endo/Twist (r^2 ^= 0.44, p = 0.005 ) and LVSD and Endo/twist (r^2 ^= 0.48, p = 0.002) has been also found.

### Discussion

According the 'Morganroth hypothesis', "the term 'athletes heart' is used to describe the characteristic enlargement (hypertrophy) of the myocardium in response to repeated exercise stimuli. Associated with this is the widely held concept that different exercise training modalities produce divergent patterns of cardiac hypertrophy in athletes [20]. Athletes in purely aerobic or endurance sports characterized by large muscle group dynamic exercise involvement (running), which subjected the LV to repetitive increase in cardiac preload exhibited 'eccentric LV hypertrophy', manifested as an increase in left ventricular (LV) cavity dimensions and a proportional increase in LV wall thickness (LVWT) to normalize myocardial strain. In contrast, resistance- or strength-trained athletes who were exposed to repetitive increases in peripheral vascular resistance and cardiac afterload, showed 'concentric LV hypertrophy', manifested as increased LVWT to normalize the increased wall tension associated with rise in pressure. Little or no effect on cavity size was apparently evident in these resistance-trained athletes" [21].

Furthermore, some sports involve heterogeneity with respect to static and dynamic cardiovascular demands in either different athletic disciplines - such as parallel bars and floor exercises in gymnastics or positions such as lineman and running back in football, or goalkeeper and mid-fielder in soccer.

However, several studies suggest that LV wall thickness is increased more in endurance than strength-trained athletes and have reported no morphological changes in resistance-trained athletes. Therefore individuals with athlete's heart could exhibit further cardiac adaptation in response to both different training and kind of practiced sport [22,23]. Although these modifications have been largely studied in elite athletes, very little informations are currently available about young elite athletes who share the same training load.

For this reasons, three groups of young athletes from different kinds of sport (cycling, soccer and basketball) and at different level of dynamic and static components, have been enrolled in the present investigation. According the sports classification [4], cyclist normally has an high dynamic and static components, soccer an high dynamic and low static component, while basketball a moderate dynamic and low static one.

LV diameter and CMi were significantly higher in cyclists compared to other groups. Although no significant differences have been found, soccer players also showed high CMi values compared to controls and basket players. Despite this, deformation parameters (strain, rotation and twist) assessed by Speckle Tracking (ST) multi-layer approach, highlighted how some differences play a relevant role distinguishing myocardial structural function.

Previous studies have shown that several physiological and hemodynamic components (as pre-load, after-load, and heart rate) are involved during the exercise. They can also be modified by age and gender [19,24]. In the present study, the basal HR in young cyclists was slightly higher respect to the other athletes. This aspect could be apparently in disagreement with a normal trend of athletes, however the obtained results can be otherwise considered substantially homogeneous in consequence of a possible not yet mature vagal tone in them.

Concerning functional aspects, a recent study investigated the effect of endurance exercise on LV torsion, showing as an increase of this parameter and also of untwisting rate playing a relevant role as components of the exercise-induced cardiac remodelling in young athletes [24].

In the present transversal investigation, evaluation by Multilayer approach of the Endocardial contribution in respect to the Endocardial one, has shown some further relevant differences. The mean *Epicardial *longitudinal strain was significantly lower in cyclists (-11.70 ± 2%) than soccer (13.43 ± 2.4%, p = .05) and basket players (13.72 ± 2.9%, p = .02), despite of mean *Endocardial *longitudinal strain value didn't show any evident difference among the groups. A trend to correlation was found in cyclists, where the CMi resulted higher either at endocardial (r^2 ^= 0.53, p = .001) or epicardial level (r^2 ^= 0.35, p = .01), while no correlation between Endo/Epi longitudinal strain and CMi was found in soccer and basketball players.

According to previous studies, a significant increase of epicardial apical rotation was found, but only in the cyclists (5.00 ± 1.7°) compared to basketball players (3.81 ± 1.5°, p = .03). This behaviour, previously demonstrated in adults [24] is reinforced in the present study by a trend to positive correlation found between both the endocardial (r^2 ^= 0.42, p < .01) and epicardial (r^2 ^= 0.27 with p = .04) apical rotation or CMi as showed in cyclists.

No evident correlation between these parameters, on the contrary, has been observed among the others groups of athletes.

All the results found suggests that there is a strong relationship in young athletes, between the myocardial remodeling and the degree of LV apical rotation, especially at epicardial level. This aspect is in accordance with previous studies regarding the longitudinal strain parameter.

The myocardial contractile function also seems to have a functional reserve in the young athletic population. Therefore the results emphasize the importance of a strain parameter and in particular the role of twist to assess regional myocardium specificities in a young trained athlete's heart.

In particular the ST method, in a multi-layer approach, can be considered a helpful tool to distinguish the Endo/Epi rotation and twist measure. This would permit a further investigation of both the subepicardium and subendocardium role of these segments in global myocardial contractility [25,26]. The obtained data also focuses on the presence of deformation parameter values within the normal and validated range in young athletes confirming a normal myocardial function. These results should be taken into serious consideration and the parameters should be used in adolescents in situations where there is clinical doubt about heart performance. Diastolic function also seems to be influenced by the remodelling structure, as all the considered parameters (E wave, A wave, IVRT) have shown. The higher reduction was found in cyclists. This preliminary aspect, in agreement with previous studies, requires a more thorough and accurate investigation with the assessment of the untwisting rate parameters.

## Conclusions

The young athlete's heart is characterized by specific, progressive adaptations to regular sport activity and training. However, in some cases (such as in cyclists) LV hypertrophy cannot be fully classified as concentric or eccentric, but represent a mixture of both types. The present results found and the increase of epicardial parameters of mechanical deformation that could be an adaptation to hard training in order to increase LV performance. The use of a speckle tracking method in a multylayer approach allows a more complete assessment of the LV chamber in adolescent agonistic athletes, representing a non-invasive method to investigate the LV performance by Endo/Epi rotation in presence of physiological myocardial hypertrophy. Considering our results and the large heterogeneity of echocardiographic pattern generally found in adolescent subjects, several possible clinical applications of this peculiar deformation parameters approach can be advised.

## Competing interests

The authors declare that they have no competing interests.

## Authors' contributions

ADL and LS have made substantial contributions to study conception and design, acquisition of data, analysis and interpretation of data. GP and GG have been involved in revising study critically and have given final approval of the version to be published. SP has been involved in revising the technical aspects of the used tools and given final approval of the version to be published. All authors read and approved the final manuscript.
